# Comparison of Rhizospheric and Endophytic Bacterial Compositions between Netted and Oriental Melons

**DOI:** 10.1128/spectrum.04027-22

**Published:** 2023-01-09

**Authors:** Jian Xiao, Yan Sun, Yi He, Xiaofu Tang, Shangdong Yang, Jinyan Huang

**Affiliations:** a Guangxi Key Laboratory of Agro-Environment and Agro-Products Safety, National Demonstration Center for Experimental Plant Science Education, Agricultural College, Guangxi University, Nanning, China; b Horticultural Research Institute, Guangxi Academy of Agricultural Sciences, Nanning, Guangxi, People’s Republic of China; c Longping Branch, College of Biology, Hunan University, Changsha, China; State Key Laboratory of Microbial Resources, Institute of Microbiology, Chinese Academy of Sciences

**Keywords:** netted melon (var. *reticulatus* Naud.), oriental melon (var. *conomon* Makino), endophytic bacteria, high-throughput sequencing, rhizosphere

## Abstract

To elucidate the biological mechanism of formation of the netted pattern in melons, the characteristics of the soil bacterial community structure in the rhizosphere and of the endophytic bacteria in the stems of netted melons were analyzed. High-throughput sequencing technology was used for the analysis of plant stem and soil samples collected from netted melons (NM) and oriental melons (OM). At the phylum level, Acidobacteria, Dependentiae, and Chloroflexi were the dominant endophytic bacteria in the stems of NM only. In addition, at the genus level, the soil bacteria enriched in the rhizospheres of NM and OM were different. Five unique dominant bacterial genera, including *Gaiella*, *Actinoplanes*, *norank_f__Gemmatimonadaceae*, *Devosia*, and *Bradyrhizobium*, were the dominant soil bacteria unique to the rhizosphere of NM. In contrast, Mycobacterium and *unclassified_f__Acetobacteraceae* were the dominant soil bacteria in the rhizosphere of OM. Moreover, *Hyphomicrobium*, *Nocardioides*, *norank_f__norank_o__Gaiellales*, *Bryobacter*, *unclassified_f__Pseudonocardiaceae*, *Pseudolabrys*, *norank_f__Micropepsaceae*, *Ideonella*, *Mizugakiibacter*, *norank_f__Vermiphilaceae*, *unclassified_f__Xanthobacteraceae*, *Bacillus*, and *Pseudaminobacter* were the dominant endophytic bacteria in the stems of NM. In contrast, *Flavobacterium*, *Stenotrophomonas*, *unclassified_f__Burkholderiaceae*, *Paenibacillus*, *Bordetella*, *Hephaestia,* and *Ideonella* were the dominant endophytic bacteria in the stems of OM. The specific substances (enzymes, proteins, endogenous hormones, etc.) secreted by unique rhizospheric and endophytic bacteria, such as *Bacillus* and *Bradyrhizobium*, may activate the promoters of genes. Therefore, the expression of genes can be regulated by unique rhizospheric and endophytic bacteria for formation or nonformation of netting in melons.

**IMPORTANCE** The study of the differential structures and functions of rhizospheric and endophytic bacterial communities between netted melon and oriental melon treatments is investigated. Our findings make a significant contribution to the literature because they are the first step in coupling the study of rhizospheric and endophytic microbial community structure to reticulation formation in netted melon. Further, we believe that this research appears to be meaningful because it provides new insights into the mechanisms of reticulation formation in netted melon in modern agricultural production.

## INTRODUCTION

Netted melon (*Cucumis melo* L. var. *raticulalus* Naud.), which belongs to the family of cucurbitaceous melons, is an edible, widely grown fruit in China (1). The fruits are called netted melons because the skin of the ripe fruit has net-shaped cracks (2). Netted melons are rich in vitamins and minerals and can quench thirst, cool the body, reduce inflammation, prevent overheating in summer, and exert a calming effect (2).

Netted melon is a popular fruit in fresh food markets due to their unique netted patterns, aesthetically pleasing shape, high sugar content, good taste, and unique flavor and pleasant aroma (3), factors which make netted melon a widely planted fruit worldwide. Currently, there are over 460,900 ha of netted melon planted in China every year (4). It is well known that reticulation is one of the important trait indicators used to characterize the quality of netted melons. However, during the production of netted melons in facilities, different sizes and roughness of the reticulated lines on the fruit surface can easily occur, as well as uneven formation of reticulation, which seriously affects their commercial appeal. Therefore, it is necessary to regulate and control the substrate water content throughout all growing stages of netted melons for quality improvement reasons (4). Many studies have found that the reticulated formation of netted melon is closely related to phytohormones, artificial influences, light, temperature, water, and other environmental conditions (5 to 7).

Studies have also confirmed that crop rhizosphere soil microbes contribute to sustainable crop production (8 to 10), such as through the mitigation of environmental stresses, drought, cold and heat damage, etc. (11 to 13). Endophytic bacteria are distributed in all parts of the plant and have functions in nitrogen fixation (14 to 15), phosphorus solubilization (16), production of plant-growth-regulating substances (17 to 18), enhancement of host resistance (19 to 20), and bioremediation (21). They therefore play important roles in regulating the microecological balance and in promoting healthy growth of their host plants (22). In addition, metagenomic studies have revealed that factors such as plant species, organ type, phenologic stage, soil type, and fertilization affect the endomicrobiomes of plants (23).

In previous field investigations, an interesting phenomenon was observed whereby different melon varieties grown under the same environmental and management conditions can be both netted and nonnetted. It is well known that the formation of reticulation in melons is controlled by genes, and the expression of genes can be affected by many factors. However, whether the different genes of melons recruit different rhizospheric or endophytic bacteria by controlling the secretion of certain substances (enzymes, proteins, hormones, etc.) had not previously been explored. Therefore, we comparatively analyzed the soil bacterial composition in the rhizospheres and endophytic bacterial communities in the stems of netted and oriental melons to explore the functional bacteria related to reticulation formation in netted melons. This will be helpful in developing functional bacterial resources and producing high-quality melons.

## RESULTS

### Community composition of soil bacteria in rhizosphere.

First, the coverage rates all reached 98% and the data were reliable (Table 1). The Shannon and Simpson indexes and the Ace and Chao1 indexes, which were used to represent the soil bacterial diversity and richness in rhizospheres of netted (NM) and oriental (OM) melons, were all significantly higher than those of bulk soils (CK) (*P < *0.05). However, they were not significantly different between NM and OM (*P > *0,05). The results suggest that the soil bacterial diversity and richness in rhizospheres of NM and OM were not significantly different (*P > *0.05) (Table 1).

**TABLE 1:** Diversity indexes of rhizosphere soil bacteria of netted melons and oriental melons^*a*^

Sample	Shannon index	Simpson index	Ace index	Chao1 index	Coverage
NM	5.94 ± 0.07 a	0.0073 ± 0.0007 b	2379.39 ± 204.29 a	2336.36 ± 142.42 a	0.98
OM	5.91 ± 0.12 a	0.0069 ± 0.0014 b	2165.85 ± 189.98 a	2167.16 ± 182.15 a	0.98
CK	5.37 ± 1.18 b	0.0245 ± 0.0301 a	1610.68 ± 624.66 b	1594.78 ± 633.43 b	0.99

a Values followed by different small letters represent significant differences between netted melons and oriental melons (*P *<* *0.05). Netted melon: NM; oriental melon: OM; bulk soil: CK.

**FIG 1:**
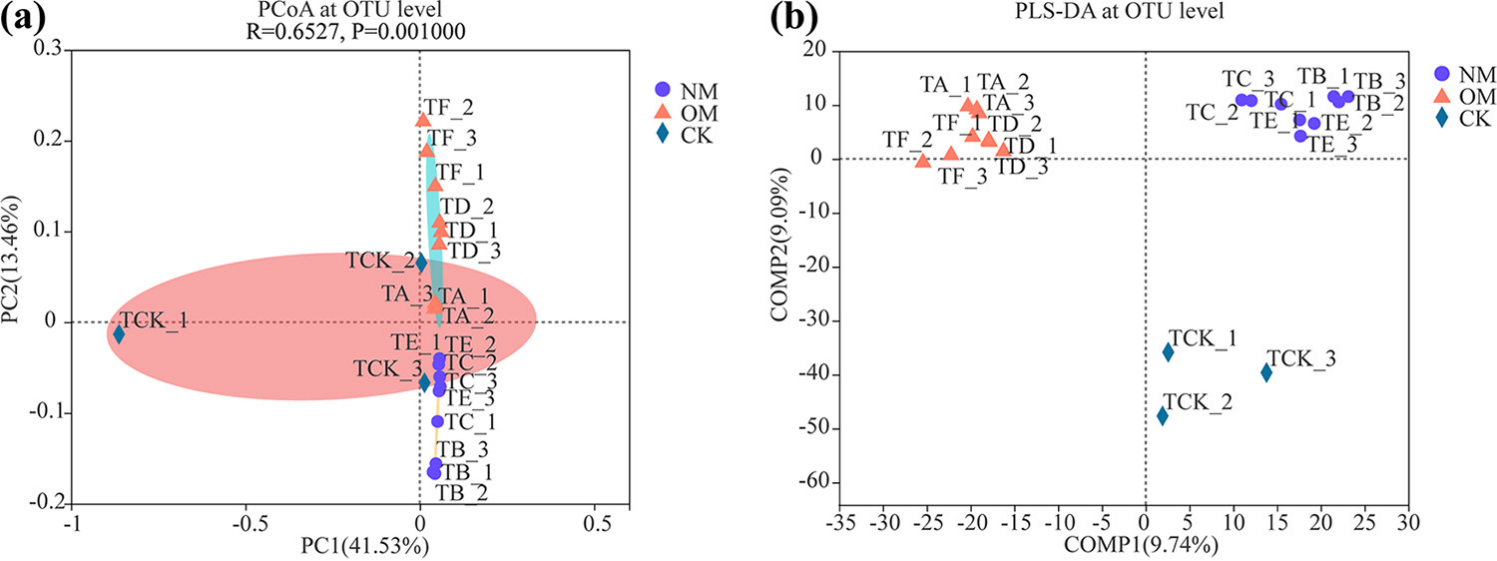
PCoA (a) and PLS-DA (b) of soil bacterial communities at the OTU level in the rhizospheres of netted melons (NM) and oriental melons (OM).

Furthermore, principal coordinate analysis (PCoA) and partial least-squares discriminant analysis (PLS-DA) at the operational taxonomic unit (OTU) level were used to evaluate the extent of the similarity of the rhizospheric bacterial communities (Fig. 1). The results showed that the rhizospheric bacterial communities of NM and OM were significantly different from CK (*P < *0.001) (Fig. 1a). The soil bacteria communities in the rhizospheres of NM, OM, and CK were separately clustered (Fig. 1b).

**FIG 2:**
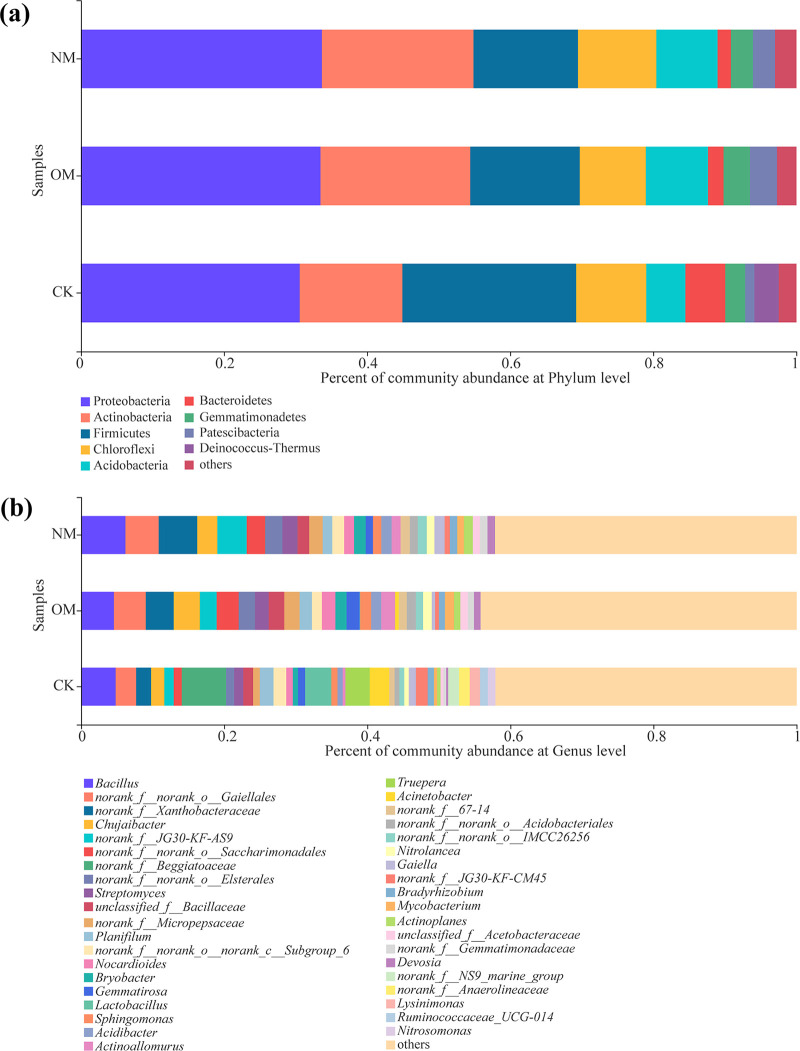
Proportions of dominant bacteria in the rhizospheres of netted (NM) and oriental (OM) melons and bulk soil (CK) at the phylum (a) and genus (b) levels. Phyla and genera representing less than 1% of the total reads are grouped as “others.”

At the phylum level, the number of dominant bacterial phyla (proportions greater than 1%) in the rhizospheres was eight for both netted and oriental melons (Fig. 2a). However, the number of dominant bacterial phyla in the background soil (CK) was nine, and their proportions from high to low were Proteobacteria (30.58%), Actinobacteria (14.35%), Firmicutes (24.28%), Chloroflexi (9.80%), Bacteroidetes (5.57%), Acidobacteria (5.47%), Deinococcus-Thermus (3.40%), Gemmatimonadetes (2.79%), Patescibacteria (1.28%), and others (2.47%).

In addition, the proportions of dominant bacterial phyla in the rhizospheres of netted melons were Proteobacteria (33.65%), Actinobacteria (21.19%), Firmicutes (14.61%), Chloroflexi (10.94%), Acidobacteria (8.56%), Gemmatimonadetes (3.12%), Patescibacteria (3.05%), Bacteroidetes (1.88%), and others (3.00%). In contrast, Proteobacteria (33.39%), Actinobacteria (21.07%), Firmicutes (15.39%), Chloroflexi (9.20%), Acidobacteria (8.66%), Patescibacteria (3.74%), Gemmatimonadetes (3.66%), Bacteroidetes (2.18%), and others (2.70%) were the dominant bacterial phyla in the rhizospheres of oriental melons. Moreover, in comparison with oriental melons, the proportions of some dominant bacterial phyla in the rhizospheres of netted melons, such as Gemmatimonadetes and Patescibacteria, were opposite to those of oriental melons (Fig. 2a).

At the genus level, the numbers of dominant bacterial genera (proportions greater than 1%) in bulk soil (CK) and rhizospheres of netted and oriental melons were 23, 27, and 24, respectively (Fig. 2b). The relative abundances of dominant bacterial genera in bulk soil (CK) and rhizospheres of netted and oriental melons are shown in Table 2. *Gaiella*, *Actinoplanes*, *norank_f__Gemmatimonadaceae*, *Devosia*, and *Bradyrhizobium* were the unique dominant bacteria in the rhizospheres of netted melons, while Mycobacterium and *unclassified_f__Acetobacteraceae* were the dominant bacteria in the rhizospheres of oriental melons (Fig. 2b).

**TABLE 2:** Proportions of dominant bacteria in the rhizospheres of netted (NM) and oriental (OM) melons and bulk soil (CK) at the genus level (%)^*a*^

Genus	CK	NM	OM
*Bacillus*	4.83	6.19	4.59
*norank_f__norank_o__Gaiellales*	2.85	4.65	4.45
*norank_f__Xanthobacteraceae*	2.09	5.38	3.91
*Chujaibacter*	1.85	2.81	3.66
*norank_f__JG30-KF-AS9*	1.32	4.10	2.34
*norank_f__norank_o__Saccharimonadales*	1.11	2.59	3.08
*norank_f__Beggiatoaceae*	6.21		
*norank_f__norank_o__Elsterales*	1.13	2.40	2.26
*Streptomyces*	1.27	2.12	1.91
*unclassified_f__Bacillaceae*	1.38	1.64	2.18
*norank_f__Micropepsaceae*		1.88	2.15
*Planifilum*	1.92	1.34	1.71
*norank_f__norank_o__norank_c__Subgroup_6*	1.79	1.66	1.39
*Nocardioides*		1.39	1.90
*Bryobacter*		1.63	1.55
*Gemmatirosa*	1.00	1.01	1.83
*Lactobacillus*	3.66		
*Sphingomonas*		1.17	1.54
*Acidibacter*		1.44	1.41
*Actinoallomurus*		1.24	1.92
*Truepera*	3.40		
Acinetobacter	2.72		
*norank_f__67-14*		1.29	1.12
*norank_f__norank_o__Acidobacteriales*		1.11	1.28
*norank_f__norank_o__IMCC26256*		1.26	1.00
*Nitrolancea*		1.08	1.22
*Gaiella*	1.00	1.43	
*norank_f__JG30-KF-CM45*	1.65		
*Bradyrhizobium*		1.03	
Mycobacterium			1.26
*Actinoplanes*		1.23	
*unclassified_f__Acetobacteraceae*			1.04
*norank_f__Gemmatimonadaceae*		1.06	
*Devosia*		1.05	
*norank_f__NS9_marine_group*	1.52		
*norank_f__Anaerolineaceae*	1.49		
*Lysinimonas*	1.44		
*Ruminococcaceae_UCG-014*	1.11		
*Nitrosomonas*	1.05		
others	42.10	42.11	44.14

a Genera representing less than 1% of the total reads are grouped as “others.”

**FIG 3:**
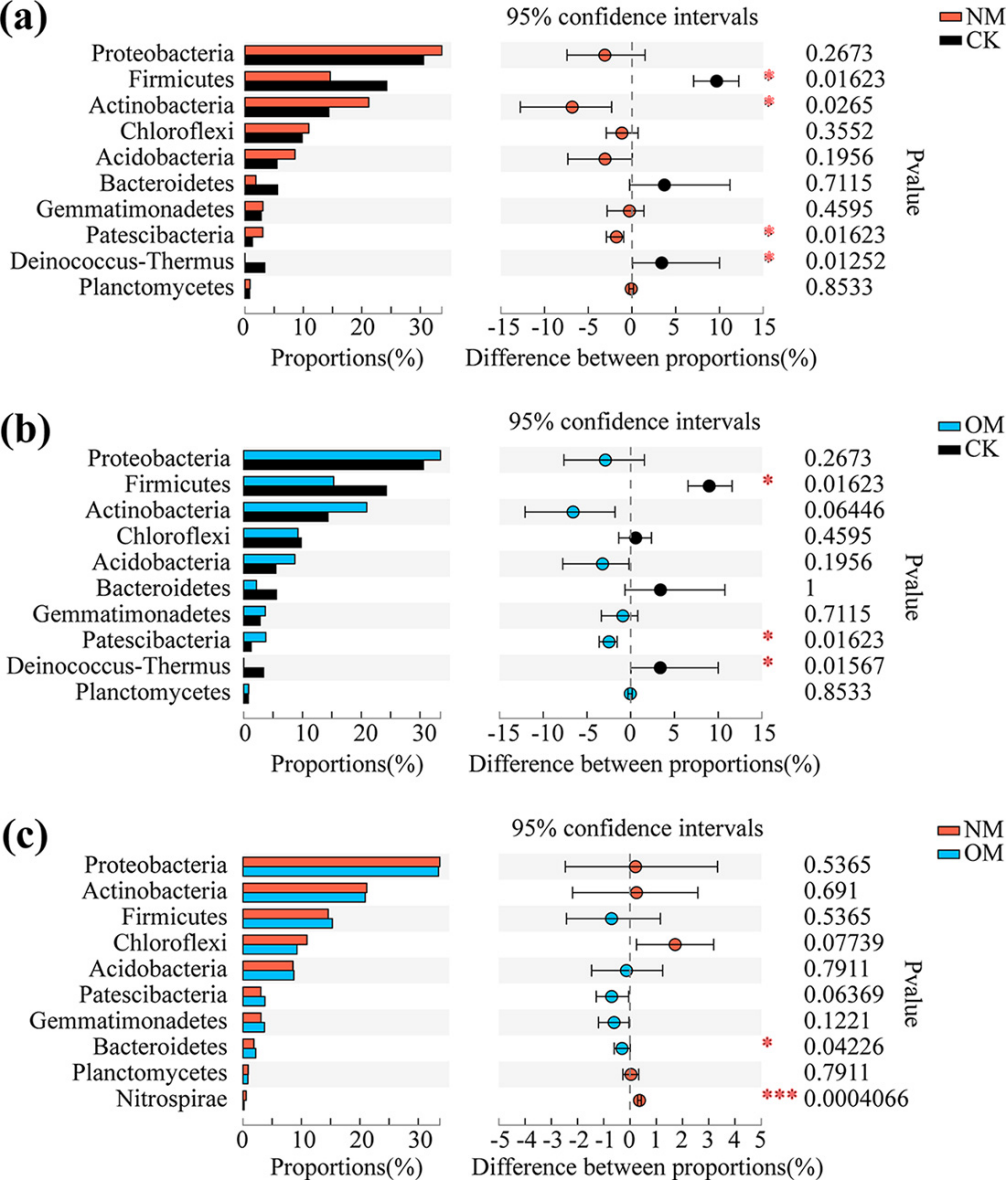
Soil bacterial phyla difference test of netted and oriental melons. *, 0.01 < *P ≤ *0.05; **, 0.001 < *P ≤ *0.01; ***, *P ≤ *0.001.

The Wilcoxon rank-sum test was used to analyze the significant differences at the phylum level of the top 10 rhizospheric soil bacteria in terms of relative abundance percentage. We found that Bacteroidetes and Nitrospirae were significantly or very significantly different in the rhizospheric soils of netted (NM) and oriental melons (OM) (Wilcoxon rank-sum test, *P < *0.05, *P < *0.001) (Fig. 3).

**FIG 4:**
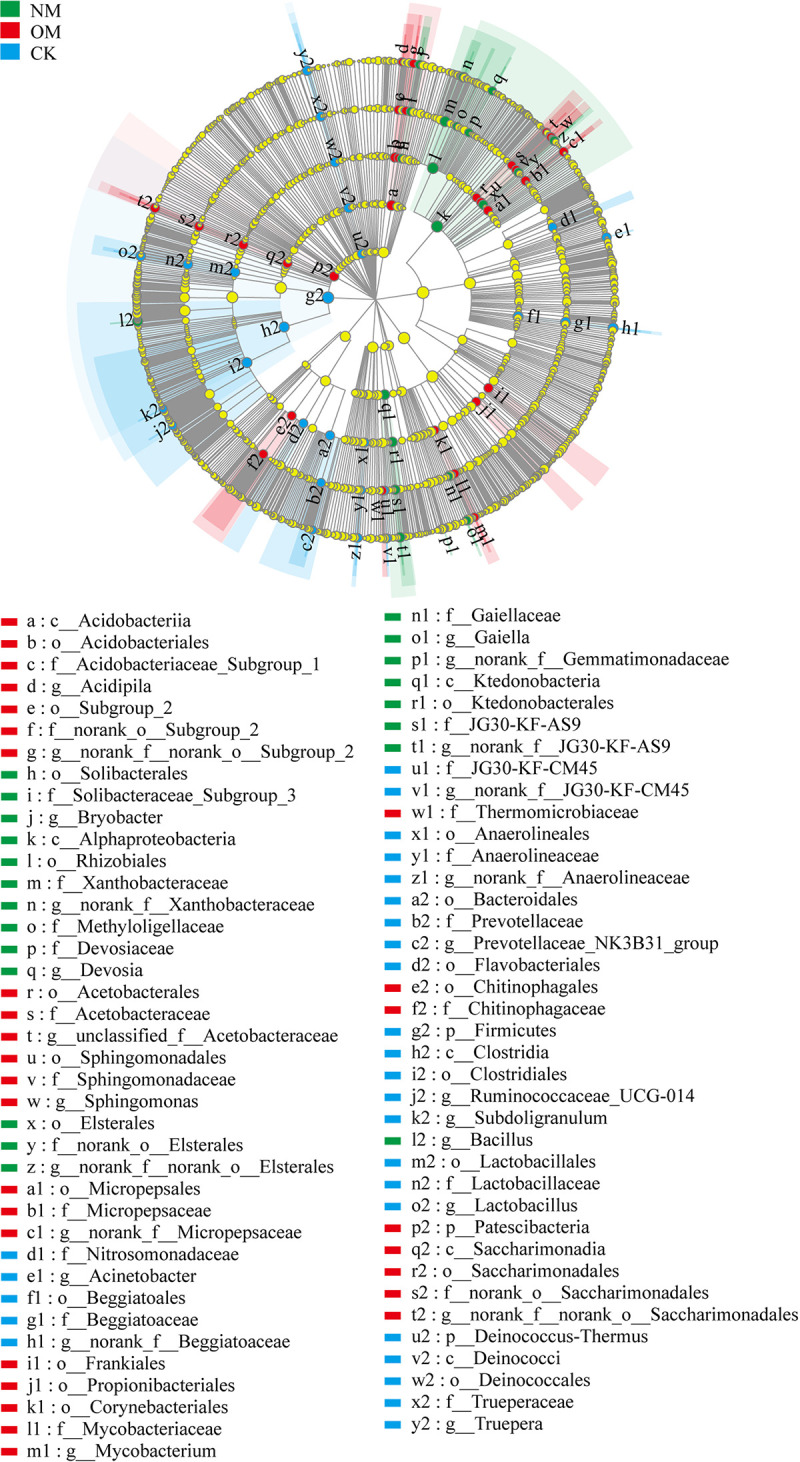
LEfSe analysis of soil bacteria in the rhizospheres of netted (NM) and oriental (OM) melons and bulk soil (CK) (*P < *0.05, LDA score = 3.5). Circles indicate phylogenetic levels from phylum to genus. The diameter of each circle was proportional to the abundance of the group. Different prefixes indicate different levels (p, phylum; c, class; o, order; f, family; g, genus).

LEfSe analysis was also conducted to identify rhizospheric bacterial responders in netted (NM) and oriental melons (OM) and CK. As seen in Fig. 4, a total of 77 rhizospheric bacterial clades exhibited significant differences (*P < *0.05, linear discriminant analysis [LDA] score > 3.5). At the genus level, *norank_f__Xanthobacteraceae*, *norank_f__JG30-KF-AS9*, *Bacillus*, *norank_f__norank_o__Elsterales*, *Bryobacter*, *Gaiella*, *Devosia*, and *norank_f__Gemmatimonadaceae* were significantly enriched in the rhizospheres of NM. In contrast, at the phylum level, only Patescibacteria was enriched, and, at the genus level, *norank_f__norank_o__Saccharimonadales*, *norank_f__Micropepsaceae*, Mycobacterium, *Acidipila*, *Sphingomonas*, *norank_f__norank_o__Subgroup_2*, and *unclassified_f__Acetobacteraceae* were significantly enriched in the rhizospheres of OM. At the phylum level, Firmicutes and Deinococcus-Thermus, and at the genus level, *norank_f__Beggiatoaceae*, *Lactobacillus*, *Truepera*, Acinetobacter, *norank_f__Anaerolineaceae*, *Ruminococcaceae_UCG-014*, *norank_f__JG30-KF-CM45*, *Prevotellaceae_NK3B31_group*, and *Subdoligranulum* were significantly enriched in CK (Fig. 4).

**FIG 5:**
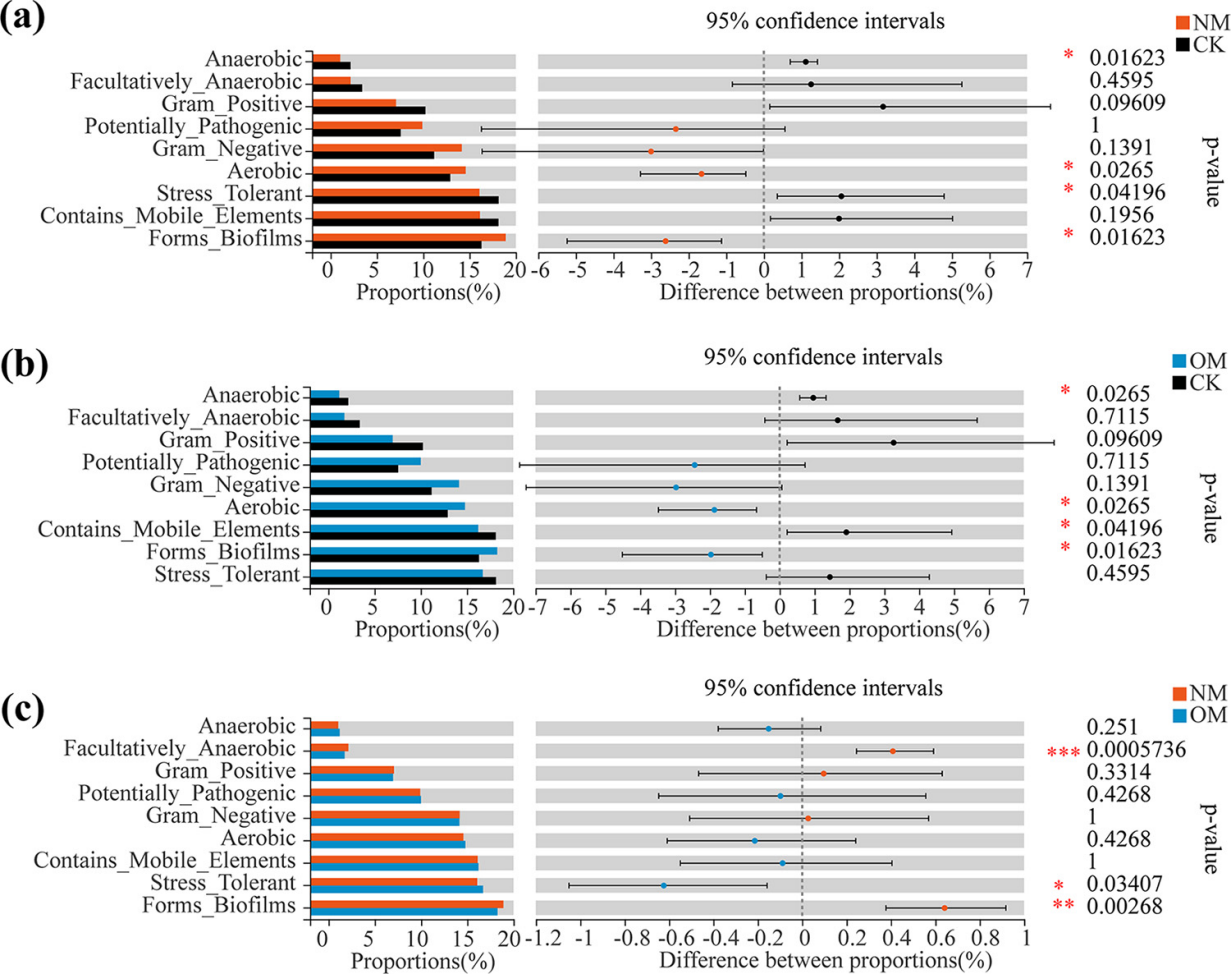
BugBase predicted phenotypic shift of soil bacterial community in the rhizospheres of netted (NM) and oriental melons (OM) and bulk soils (CK). *, 0.01 < *P ≤ *0.05; **, 0.001 < *P ≤ *0.01; ***, *P ≤ *0.001 (Wilcoxon rank-sum test).

Based on 16S rRNA gene sequences, the phenotypic shift of the bacterial community was investigated using BugBase (https://bugbase.cs.umn.edu/index.html). The relative abundance of the Facultatively Anaerobic and Forms Biofilms bacterial group in netted melon (NM) was significantly higher than in oriental melon (OM) (Fig. 5c). In contrast, the relative abundance of Stress Tolerant bacteria was significantly lower in NM compared to OM (Fig. 5).

### Community composition of endophytic bacteria in stems.

In addition, the Shannon and Simpson indexes of endophytic bacteria in the stems of netted melons were significantly higher or lower than those of oriental melons. Moreover, the Ace and Chao1 indexes of endophytic bacteria in the stems of netted melons were also significantly higher than those of oriental melons. These results suggest that the endophytic bacterial diversity and richness in the stems of netted melons were significantly higher than those of oriental melons (Table 3). In other words, it could be inferred that netted melons need to recruit or enrich more abundant endophytic bacteria for net formation. Moreover, these results also indicate that endophytic bacteria greatly contribute to net formation in melons compared to soil bacteria in the rhizosphere.

**TABLE 3:** Diversity indexes of endophytes in the stems of netted melons (NM) and oriental melons (OM)^*a*^

Sample	Shannon index	Simpson index	Ace index	Chao1 index	Coverage
NM	4.39 ± 0.15 a	0.0346 ± 0.0179 b	569.18 ± 32.92 a	570.01 ± 35.25 a	0.99
OM	3.57 ± 0.68 b	0.1022 ± 0.0843 a	488.42 ± 66.99 b	458.51 ± 78.14 b	0.99

a Values followed by different small letters represent significant differences between netted melons and oriental melons (*P *<* *0.05).

**FIG 6:**
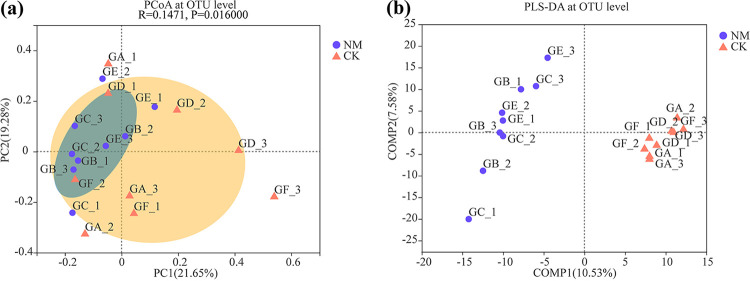
PCoA (a) and PLS-DA (b) of endophytic bacterial communities at the OTU level in the stems of netted (NM) and oriental melons (OM).

PCoA and PLS-DA at the OTU level were also used to evaluate the extent of the similarity of the endophytic bacterial communities (Fig. 6). The results showed that the endophytic bacteria communities of NM and OM were clustered separately. This means that the endophytic bacterial communities showed significant differences between NM and OM (*P < *0.05).

**FIG 7:**
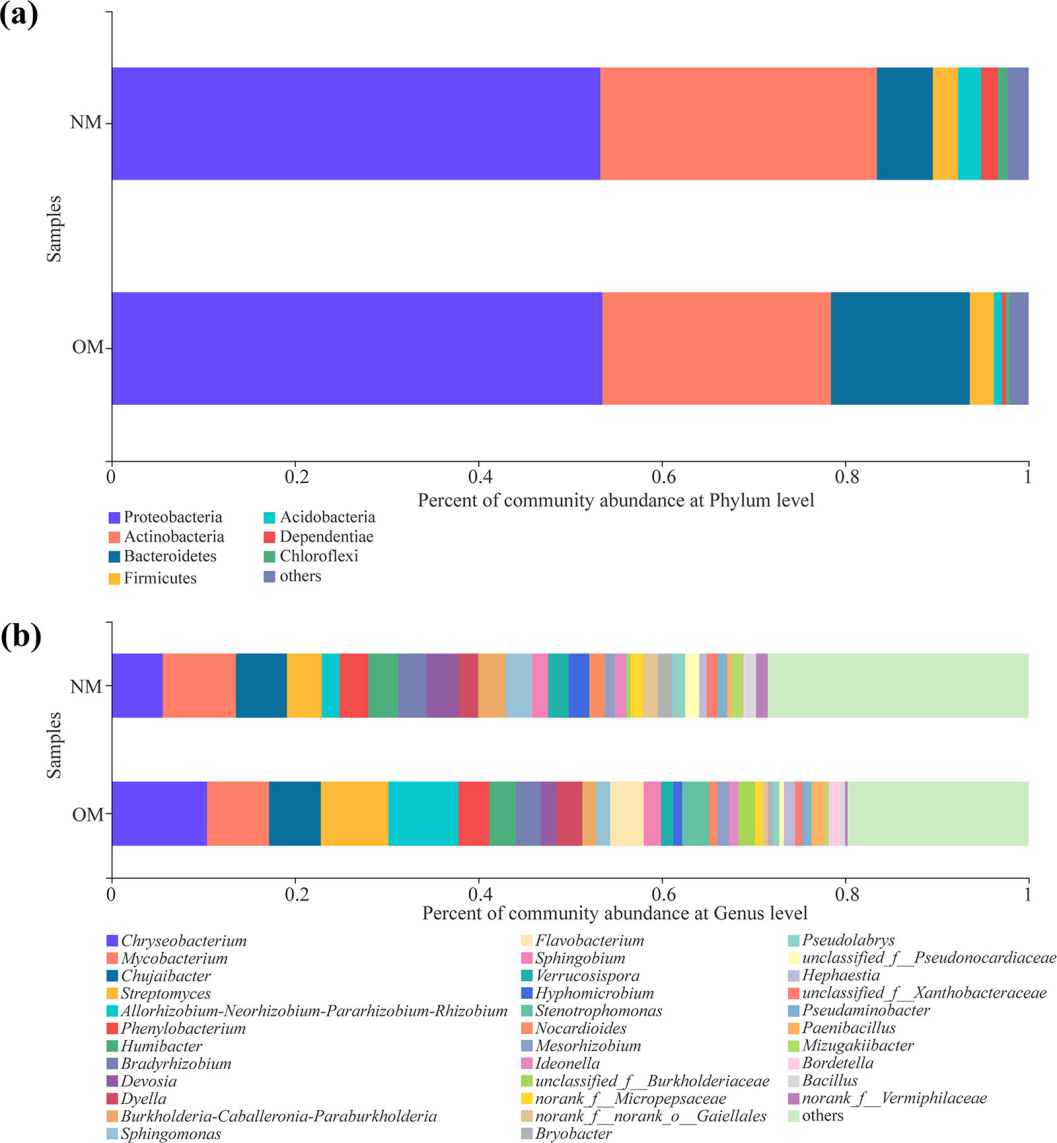
Proportions of dominant endophytic bacteria in the stems of netted (NM) and oriental (OM) melons at the phylum (a) and genus (b) level. Phyla and genera representing less than 1% of the total reads are grouped as “others.”

The number of dominant endophytic bacterial phyla (proportions greater than 1%) in the stems of NM was seven. In contrast, only four dominant endophytic bacterial phyla were detected in the stems of OM. Proteobacteria (53.30%), Actinobacteria (30.18%), Bacteroidetes (6.09%), Firmicutes (2.74%), Acidobacteria (2.55%), Dependentiae (1.82%), Chloroflexi (1.04%), and others (2.29%) were the dominant endophytic bacterial phyla in the stems of NM. In contrast, Proteobacteria (53.53%), Actinobacteria (24.92%), Bacteroidetes (15.14%), Firmicutes (2.62%), and others (2.07%) were the dominant endophytic bacterial phyla in the stems of OM. Acidobacteria, Dependentiae, and Chloroflexi were the unique dominant endophytic bacterial phyla in the stems of NM (Fig. 7a).

Moreover, at the genus level, the numbers of dominant endophytic bacteria (proportions greater than 1%) in the stems of netted and oriental melons were 28 and 22, respectively (Fig. 7b). The relative abundances of dominant endophytic bacterial genera in the stems of netted and oriental melons are shown in Table 4. *Hyphomicrobium*, *Nocardioides*, *norank_f__norank_o__Gaiellales*, *Bryobacter*, *unclassified_f__Pseudonocardiaceae*, *Pseudolabrys*, *norank_f__Micropepsaceae*, *Ideonella*, *Mizugakiibacter*, *norank_f__Vermiphilaceae*, *unclassified_f__Xanthobacteraceae*, *Bacillus*, and *Pseudaminobacter* were the unique dominant endophytic bacterial genera in the stems of NM. On the contrary, *Flavobacterium*, *Stenotrophomonas*, *unclassified_f__Burkholderiaceae*, *Paenibacillus*, *Bordetella*, *Hephaestia*, and *Ideonella* were the unique dominant endophytic bacterial genera in the stems of OM (Fig. 7b).

**TABLE 4:** Proportions of dominant endophytic bacteria in the stems of netted (NM) and oriental (OM) melons at the genus level (%)^*a*^

Genus	NM	OM
*Chryseobacterium*	5.59	10.41
Mycobacterium	7.97	6.77
*Chujaibacter*	5.56	5.65
*Streptomyces*	3.80	7.37
*AllorhizobiumNeorhizobium-Pararhizobium-Rhizobium*	1.93	7.64
*Phenylobacterium*	3.14	3.35
*Humibacter*	3.27	2.89
*Bradyrhizobium*	3.11	2.74
*Devosia*	3.50	1.68
*Dyella*	2.10	2.84
*Burkholderia-Caballeronia-Paraburkholderia*	3.04	1.53
*Sphingomonas*	2.81	1.50
*Flavobacterium*		3.66
*Sphingobium*	1.75	1.90
*Verrucosispora*	2.25	1.34
*Hyphomicrobium*	2.22	
*Stenotrophomonas*		2.98
*Nocardioides*	1.67	
*Mesorhizobium*	1.07	1.29
*Ideonella*	1.29	1.02
*unclassified_f__Burkholderiaceae*		1.80
*norank_f__Micropepsaceae*	1.37	
*norank_f__norank_o__Gaiellales*	1.62	
*Bryobacter*	1.56	
*Pseudolabrys*	1.39	
*unclassified_f__Pseudonocardiaceae*	1.54	
*Hephaestia*		1.22
*unclassified_f__Xanthobacteraceae*	1.20	
*Pseudaminobacter*	1.02	
*Paenibacillus*		1.46
*Mizugakiibacter*	1.29	
*Bordetella*		1.38
*Bacillus*	1.13	
*norank_f__Vermiphilaceae*	1.26	
Others	28.45	19.73

a Genera representing less than 1% of the total reads are grouped as “others.”

**FIG 8:**
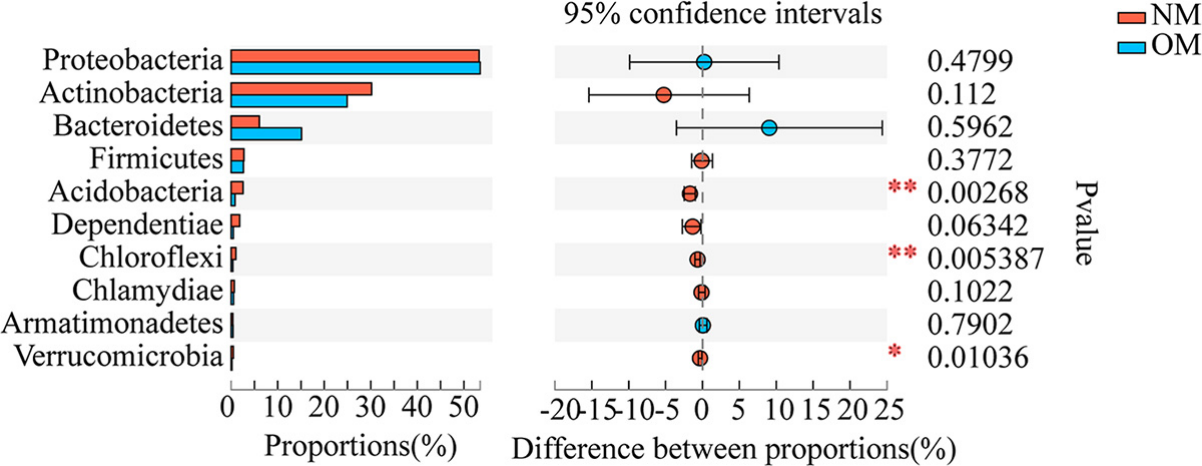
Endophytic bacterial phylum level difference test of netted and oriental melons. *, 0.01 < *P ≤ *0.05; **, 0.001 < *P ≤ *0.01; ***, *P ≤ *0.001.

The Wilcoxon rank-sum test was also used to analyze the significant differences at the phylum level for the top 10 endophytic bacteria in terms of relative abundance percentage. Among them, we found that Acidobacteria, Chloroflexi, and Verrucomicrobia were significantly or very significantly different in the stems of netted and oriental melons (Wilcoxon rank-sum test, *P < *0.05, *P < *0.01) (Fig. 8).

**FIG 9:**
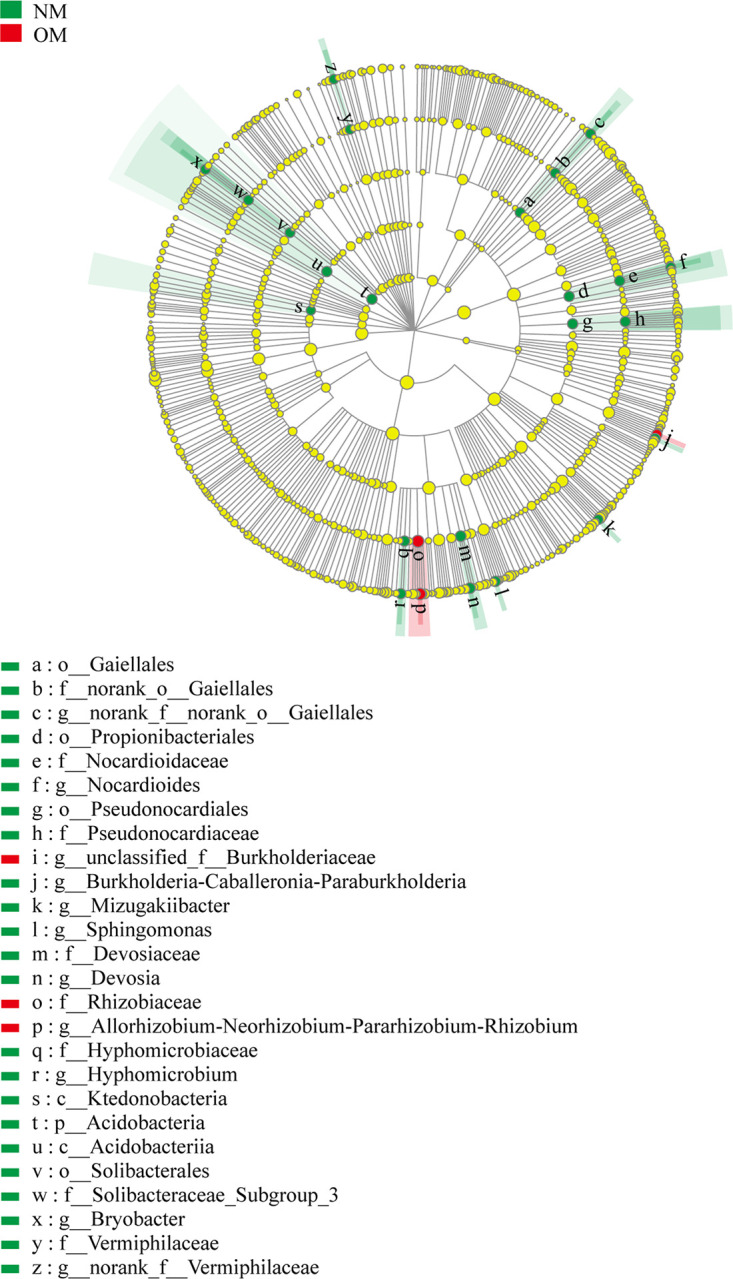
LEfSe analysis of endophytic bacteria in the stems of netted (NM) and oriental (OM) melons (*P < *0.05, LDA score = 3.5). Circles indicate phylogenetic levels from phylum to genus. The diameter of each circle is proportional to the abundance of the group. Different prefixes indicate different levels (p, phylum; c, class; o, order; f, family; g, genus).

LEfSe analysis was also conducted to identify endophytic bacterial responders for netted (NM) and oriental melons (OM). As shown in Fig. 9, a total of 26 endophytic bacterial clades exhibited significant differences (*P < *0.05, LDA score > 3.5). At the phylum level, only Acidobacteria, and at the genus level, *Devosia*, *Burkholderia-Caballeronia-Paraburkholderia*, *Sphingomonas*, *Bryobacter*, *Hyphomicrobium*, *norank_f__Vermiphilaceae*, *norank_f__norank_o__Gaiellales*, *Mizugakiibacter*, and *Nocardioides* were significantly enriched in the stems of NM. In contrast, *Allorhizobium-Neorhizobium-Pararhizobium-Rhizobium*, and *unclassified_f__Burkholderiaceae* were significantly enriched in the stems of OM (Fig. 9).

**FIG 10:**
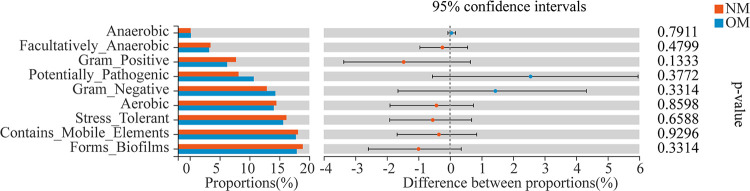
BugBase predicted phenotype shift of the endophytic bacterial community in the stems of netted (NM) and oriental melons (OM). *, 0.01 < *P ≤ *0.05; **, 0.001 < *P ≤ *0.01; ***, *P ≤ *0.001 (Wilcoxon rank-sum test).

Based on 16S rRNA gene sequences, BugBase was used to investigate the phenotypic shift of the endophytic bacterial community. The results showed that there was no significant phenotypic shift in endophytic bacterial abundance in the stems between NM and OM (Fig. 10).

## DISCUSSION

The external and internal phenotypes of netted melons, such as surface netting, color, and nutrient content, are easily affected by environmental factors (1). Previous studies have shown that netted melon external phenotypes (i.e., fruit color and skin netting) and internal phenotypes are closely related (1, 24, 25). In addition, growing netted melons requires strict cultivation conditions and complex management strategies, particularly in terms of the water status of the cultivation substrate (26 to 27). Inadequate substrate water status leads to yield loss, poor quality, low sugar content, and unaesthetic netted patterns (28 to 30). Dogan et al. found that cultivation substrate water content served as a critical factor influencing sugar accumulation and specifically netted pattern formation (31).

Suberization of artificially wounded tissues in netted melon fruit is often associated with peroxidase (POD) activity, which can polymerize phenolic monomers to generate a matrix of suberin polymer (32). Ethylene is considered to be involved in the development of lignified cell walls containing suberin polymers (33) through the activation of POD activity (34). Moreover, the peroxidase activities in three varieties of smooth-rind melon (Cucumismelo var. *inodorous*) were also found to be lower than those in the rinds of netted varieties (35).

Rhizosphere microorganisms are closely related to crop health, and changes in their community structure will directly affect the growth of crops (36). Endophytic bacteria also play an important role in promoting plant growth. Moreover, endophytic bacteria can effectively enhance disease control (37). In this study, even though soil bacterial diversity and richness in the rhizosphere were not significantly different between netted and oriental melons, the endophytic bacterial diversity and richness in the stems of netted melons were significantly higher than those of oriental melons. Therefore, it can be inferred that endophytic bacteria in stems are more closely related to reticulation formation of melon fruits than soil bacteria in the rhizospheres.

There were no specific dominant soil bacterial phyla in the rhizospheres of NM. However, at the genus level, some dominant bacterial genera, such as *Gaiella*, *Actinoplanes*, *norank_f__Gemmatimonadaceae*, *Devosia*, and *Bradyrhizobium*, were enriched, but Mycobacterium and *unclassified_f__Acetobacteraceae* were absent from the rhizospheres of NM. *Bradyrhizobium* has been reported to be involved in the production of indole-3-acetic acid (IAA) (38). Boiero et al. also confirmed that *Bradyrhizobium* can increase the content of plant endogenous ethylene (39). This indicates that the enrichment of *Bradyrhizobium* in the rhizospheres of NM could provide more phytohormones to the plants. Based on the results of Chen, a higher content of ethylene in plants could be considered one of the important reasons for the formation of reticulation in reticulated melon fruits (5).

In addition, Acidobacteria, Dependentiae, and Chloroflexi were three specific dominant endophytic bacterial phyla enriched in the stems of netted melons. A previous study found that Chloroflexi bacteria tend to live in a nutrient-rich environment, and that large amounts of nutrients favor its growth and reproduction (40). The formation of netted melon reticulation is correlated with the concentration of Ca^2+^ (41). Interestingly, Hugoni et al. also found that Leotiomycetes and Firmicutes were correlated with Ca^2+^ (42). In our study, we found that the relative abundance of endophytic Firmicutes was higher in the stems of NM compared to the stems of OM. Meanwhile, in comparison with oriental melons, *Hyphomicrobium*, *Nocardioides*, *norank_f__norank_o__Gaiellales*, *Bryobacter*, *unclassified_f__Pseudonocardiaceae*, *Pseudolabrys*, *norank_f__Micropepsaceae*, *Ideonella*, *Mizugakiibacter*, *norank_f__Vermiphilaceae*, *unclassified_f__Xanthobacteraceae*, *Bacillus*, and *Pseudaminobacter* were the unique dominant endophytic bacterial genera in the stems of netted melons. In contrast, *Flavobacterium*, *Stenotrophomonas*,*unclassified_f__Burkholderiaceae*, *Paenibacillus*, *Bordetella*, *Hephaestia*, and *Ideonella* were the dominant endophytic bacterial genera in the stems of oriental melons.

Not only can *Bacillus* effectively induce enhanced POD activity, but it also has a role in promoting phosphorus uptake (43 to 44). Kang et al. also revealed that either the phytohormone (GAs and IAA) secretion or phosphate solubilization ability of *Bacillus* species enhances plant growth (45). *Bacillus* is enriched as a unique endophytic bacterial genus in the stems of NM; it can be inferred that more abundant phytohormones and an environment with higher peroxidase activity may be responsible for reticulation formation on the fruit surface of NM. The expression of genes can be adjusted as dominant or recessive by the activation of promoters, and promoters are composed of specific proteins, enzymes, endogenous hormones, etc. These specific substances are mostly secreted by endophytic or rhizospheric bacteria among melons with different genotypes.

### Conclusion.

The compositions of the soil bacterial communities in the rhizospheres and endophytic bacterial communities in the stems of netted and oriental melons were analyzed. The conclusions are as follows:

(i) There was no significant difference in soil bacterial diversity and richness in the rhizospheres of netted and oriental melons. However, the endophytic bacterial diversity and richness in the stems of netted melons were significantly higher than those of oriental melons. The functions of endophytic bacteria in contributing to reticulation formation of melons could be more significant than those of the soil bacteria in the rhizospheres.
(ii) Compared to oriental melons, although there were no specific dominant bacterial phyla in the rhizospheres of netted melon, *Gaiella*, *Actinoplanes*, *norank_f__Gemmatimonadaceae*, *Devosia,* and *Bradyrhizobium* were enriched as the unique dominant bacterial genera in the rhizospheres of netted melons. In addition, *Hyphomicrobium*, *Nocardioides*, *norank_f__norank_o__Gaiellales*, *Bryobacter*, *unclassified_f__Pseudonocardiaceae*, *Pseudolabrys*, *norank_f__Micropepsaceae*, *Ideonella*, *Mizugakiibacter*, *norank_f__Vermiphilaceae*, *unclassified_f__Xanthobacteraceae*, *Bacillus,* and *Pseudaminobacter* were the unique dominant endophytic bacterial genera in the stems of netted melons.

The above results suggest that melons with different genotypes, particularly netted melons, need to recruit more abundant specific endophytic or rhizospheric bacteria for reticulation formation on the fruit surface.

## MATERIALS AND METHODS

### Study site description.

The experiment was conducted at the experimental base of the Agricultural College of Guangxi University (108°17′E, 22°51′N). The soil physicochemical properties in the field of the experimental base were as follows: soil pH 5.68, organic matter content 8.92 g·kg^−1^, and total nitrogen, phosphorus, and potassium 0.55 g·kg^−1^, 0.67 g·kg^−1^, and 7.51 g·kg^−1^, respectively. The available nitrogen, phosphorus, and potassium were 15.27 mg·kg^−1^, 0.67 mg·kg^−1^, and 82.8 mg·kg^−1^, respectively.

### Test material.

**FIG 11:**
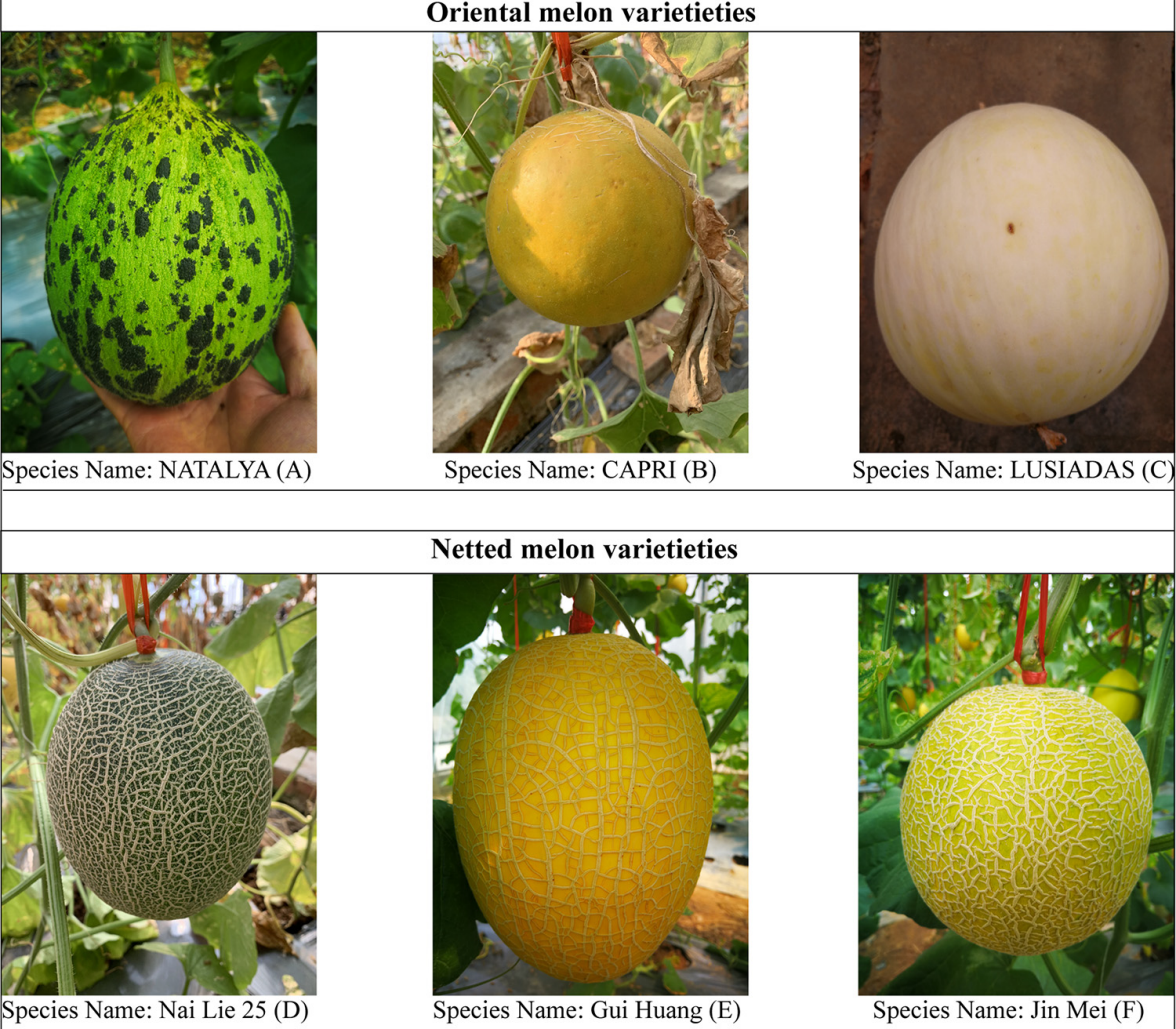
The appearance and morphological characteristics of different *Cucumismelo* L varieties.

The oriental melon (OM) varieties NATALYA (A), CAPRI (B), and LUSIADAS (C) (Fig. 11A to C), and the netted melon (NM) varieties Nai Lie 25 (D), Gui Huang (E), and Jin Mei (F) (Fig. 11D to F) (purchased from Newneme (Beijing) Seed Co. (Fig. 11A to F), were used in this experiment. Plants were all grown to seedling stage and planted in the same field on 9 June 2019, under identical management conditions. Briefly, seedlings of each melon variety with three true leaves were planted in lines at a distance of 50 cm apart on 120-cm beds in a greenhouse from 2017 to 2019 in spring and autumn, respectively. In every year, each melon variety was replicated three times, and the management practices on each melon variety were carried out identically following the previous study (7).

### Soil and plant sample collection.

Soil and plant samples were collected when the fruits were ripened for harvesting. Bulk soils with no melon growing in them in melon-growing fields were collected randomly for the soil background values (CK). Rhizosphere soils from every melon variety were collected randomly using the shake method described by Riley and Barber (46). Briefly, a circle with a radius of about 25 cm was shoveled loose with a sterilized shovel at the center of the plant and the whole melon plant was pulled out by hand, holding the base of the plant stem, and then the soil attached to the roots was forcefully shaken off and carefully collected for the rhizosphere soil samples. The soil samples were passed through a 2-mm sieve and stored at −80°C for DNA extraction (47).

At the same time, stem samples were collected separately from the plants using sterilized scissors. These were placed in sealed sterile bags and labeled, and were rinsed and wiped for 2 min with sterile water and a soft brush to remove the surface impurities and adherents from the stems of the melons. The stem samples were washed in 75% ethanol for 1 min, then 1% NaClO solution for 3 min, and finally the plant stems were washed with sterile water for 0.5 min and sterile paper was used to remove water from the stem surface of the melons (48). To determine the success of sterilization of the melon stem surface, 100 μL water from each washed stem was placed on a Luria-Bertani (LB) agar plate (g/L) (NaCl-10, tryptone-5, yeast extract-5, and agar-20) and incubated at 25°C for 7 days. No colonies developed on the plates, confirming that they were thoroughly sterilized. The sterilization of the stem surface samples was completed before detection and analysis of the endophytic microorganisms (49). The stems were placed in sterile bags and stored at −80°C for DNA extraction.

### Analysis of rhizospheric soil and endophytic bacterial compositions.

The total DNA extraction, PCR amplification, and sequence determination were carried out sequentially, following previous protocols (47).

Microbial community genomic DNA was extracted from samples using the E.Z.N.A. DNA kit (Omega Bio-tek, Norcross, GA, USA) according to the manufacturer’s instructions. The DNA extract was checked on a 1% agarose gel, and the DNA concentrations and purity were determined using a NanoDrop 2000 UV–Vis spectrophotometer (Thermo Scientific, Wilmington, USA). PCR amplification and sequencing of the total DNA extracted from the plant stem samples were performed by Shanghai Majorbio Bio-Pharm Technology Co., Ltd. The hypervariable V3–V4 region of the bacterial 16S rRNA gene was amplified using primer pairs 338F (5′-ACTCCTACGGGAGGCAGCAG-3′) and 806R (5′-GGACTACHVGGGTWTCTAAT-3′). The primers 799F (5′-AACMGGATTAGATACCCKG-3′) and 1392R (5′-ACGGGCGGTGTGTRC-3′) were selected for the first round of PCR amplification of the V5–V7 variable region, and the primers 799F (5′-AACMGGATTAGATACCCKG-3′) and 1193R (5′-ACGTCATCCCCACCTTCC-3′) were selected for the second round of PCR amplification of the V5–V7 variable region. PCR amplification was performed on an ABI GeneAmp 9700 PCR thermocycler (ABI, CA, USA), and the PCR products were recovered using 2% agar gel electrophoresis. The products were purified using an AxyPrep DNA Gel Extraction kit (Axygen, USA) and quantified using a Quantus Fluorometer (Promega, USA). The purified amplicons were pooled in equimolar quantities and were paired-end sequenced (2 × 300) on the Illumina MiSeq platform (Illumina, San Diego, USA), according to the standard protocols of Majorbio Bio-Pharm Technology Co., Ltd. (Shanghai, China). The processing and analysis of sequencing data have been described previously in detail (47, 50 to 52).

### Statistical analyses.

The experimental data were analyzed using Excel 2019 and IBM SPSS Statistics 21, and the results are shown as means with their standard deviations (mean ± SD). Mothur (version 1.30.2) was used to calculate alpha diversities of the microbial communities. Principal coordinate analysis (PCoA) was performed, and the R language (version 3.3.1) tool was used for statistical analysis and graphing. The mixOmics package was used and graphed for Partial Least Squares Discriminant Analysis (PLS-DA) using the R language (version 3.3.1) tool. OTU tables with a 97% similarity level were selected for microbial community composition and Venn diagram analysis, and the R language (version 3.3.1) tool was used for statistics and graphing. LEfSe was used to perform linear discriminant analysis (LDA) on samples according to different grouping conditions based on taxonomic composition, to identify clusters that had a significant differential impact on sample delineation. BugBase (https://bugbase.cs.umn.edu/index.html) was used for phenotypic prediction. The Majorbio Cloud Platform (www.majorbio.com) of Majorbio Bio-Pharm Technology Co., Ltd. (Shanghai, China) was used to conduct online data analysis (51 to 52).

### Data availability.

Raw reads were deposited in the NCBI Sequence Read Archive (SRA) database (accession number: PRJNA881489).
